# Loss of genetic integrity and biological invasions result from stocking and introductions of *Barbus barbus*: insights from rivers in England

**DOI:** 10.1002/ece3.1906

**Published:** 2016-01-28

**Authors:** Caterina Maria Antognazza, Demetra Andreou, Serena Zaccara, Robert J. Britton

**Affiliations:** ^1^ Department of Life and Environmental SciencesBournemouth UniversityBH12 5BBPooleDorsetUK; ^2^ Dipartimento di Scienze Teoriche e ApplicateUniversità degli Studi dell'Insubria21100VareseItaly

**Keywords:** European barbel, genetic differentiation, indigenous, invasive, nonindigenous

## Abstract

Anthropogenic activities, including the intentional releases of fish for enhancing populations (stocking), are recognized as adversely impacting the adaptive potential of wild populations. Here, the genetic characteristics of European barbel *Barbus barbus* were investigated using 18 populations in England, where it is indigenous to eastern‐flowing rivers and where stocking has been used to enhance these populations. Invasive populations are also present in western‐flowing rivers following introductions of translocated fish. Two genetic clusters were evident in the indigenous range, centered on catchments in northeast and southeast England. However, stocking activities, including the release of hatchery‐reared fish, have significantly reduced the genetic differentiation across the majority of this range. In addition, in smaller indigenous rivers, populations appeared to mainly comprise fish of hatchery origin. In the nonindigenous range, genetic data largely aligned to historical stocking records, corroborating information that one particular river (Kennet) in southeast England was the original source of most invasive *B. barbus* in England. It is recommended that these genetic outputs inform management measures to either restore or maintain the original genetic diversity of the indigenous rivers, as this should help ensure populations can maintain their ability to adapt to changing environmental conditions. Where stocking is considered necessary, it is recommended that only broodstock from within the catchment is used.

## Introduction

In this era of rapid environmental change, local adaptation processes and the adaptive potential of wild populations are important to conserve as they potentially provide populations with inherent resilience to the disturbed conditions (Jensen et al. 2008). For example, adaptive capacity is important in the context of climate change where populations must either adapt to the altered conditions via plastic changes or their population must undergo evolutionary adaptation (Hoffmann and Sgro 2011). Moreover, anthropogenic activities are increasingly recognized as impacting upon the local adaptation and adaptive potential of populations, with factors such as habitat loss and introductions of alien species recognized as playing major roles in reducing the genetic capacity of populations to respond to environmental changes (Bijlsma and Loeschcke 2011; Sgro et al. 2011).

In freshwaters, salmonid fishes are generally recognized as having strong patterns of genetic differentiation, with populations showing strong adaptation to local rivers (e.g., Griffiths et al. 2009). However, salmonids are also one of the most artificially reared and stocked family of fishes in the world with, for example, 1.7 billion fish (mainly *O. mykiss*) released into the wild in the USA in 2004, a rate considered as low compared with 1951 to 2000 (Halverson 2008). This is important, given that fish translocation, stocking, and introduction activities are increasingly recognized as being detrimental to wild conspecifics in the receiving waters. For example, populations subjected to regular fish stockings tend to have reduced genetic diversity (Eldridge et al. 2009), lack genetic differentiation with other populations (Sušnik et al. 2004; Eldridge and Naish 2007; Perrier et al. 2013), and their local gene pools are displaced (Laikre et al. 2010). This can negatively impact the extant population's genetic integrity (Laikre et al. 2010) and evolutionary potential (Araki et al. 2008; Mclure et al. 2008), making local populations less suited to their environment in the long‐term. There is also little knowledge on how the genetic composition of the populations evolves once stocking has ceased (Hansen et al. 2010; Perrier et al. 2013).

In England, there has also been a strong emphasis on using salmonid fishes to enhance recreational fisheries (Aprahamian et al. 2003, 2004), usually using *O. mykiss* (Fausch 2007). In lowland areas, however, recreational angling is based more on catch and release angling for species of the Cyprinidae family. From the latter part of the 19th century and up to the present, this has resulted in large numbers of cyprinid fishes being moved between river catchments to enhance and/or create fisheries (e.g., Wheeler and Jordan 1990), heavily impacting their natural distributions. More recently, it has also involved the rearing of fishes in designated hatcheries for subsequent release into the wild (at least 250,000 fish per year; Britton et al. 2004, 2008). Despite the socioeconomic value of their fisheries and the large numbers of fish moved between basins, there is scant information on the genetic composition of native cyprinid fishes in England, and the evolutionary and adaptation consequences of their stocking and introduction activities, such as genetic drift, dilution of wild gene pools, and loss of genetic diversity.

A species frequently used to create and enhance recreational fisheries in England, and elsewhere in Europe, is European barbel *Barbus barbus* (Britton and Pegg 2011). Biogeographically, populations in England are indigenous only to eastern‐flowing rivers (Wheeler and Jordan 1990). Since the 1890s, however, there have been introductions of translocated fish into a high proportion of western‐flowing rivers (Wheeler and Jordan 1990; Britton and Gozlan 2013). Historically, this involved the movement of mature fish from indigenous to nonindigenous rivers; more recently, it has relied primarily on releasing hatchery‐reared, juvenile fish (Wheeler and Jordan 1990; Britton and Gozlan 2013). Thus, they provide a novel opportunity to investigate the genetic consequences of human mediated movements of cyprinid fishes in a defined spatial range and whose outputs will have evolutionary significance and application to developing informed conservation and fishery management strategies. Consequently, the objectives of this study were to (1) assess the genetic diversity of *B. barbus* populations in indigenous river catchments in England and the extent to which stocking has impacted genetic integrity and differentiation between river catchments, (2) reconstruct introduction patterns in nonindigenous river basins and identify the genetic source of successful invasive populations, and (3) evaluate the evolutionary significance of these outputs and their potential applications for improving the management of their populations.

## Materials and Methods

### *Barbus barbus* in England and Wales

*Barbus barbus* is the only *Barbus* species present in England and Wales and thus translocated fish pose no risk of hybridisation with endemic *Barbus* species as has occurred elsewhere in Europe (Meraner et al. 2013; Zaccara et al. 2014). The indigenous range of *B. barbus* in England covers the Yorkshire Ouse, Trent, and Thames river basins (Table 1; Wheeler and Jordan 1990). Although there a number of other eastern‐flowing rivers that could theoretically have also held natural stocks, there is some doubt over whether this would be the case, as these rivers are generally small and habitat limiting, such as the Wensum, Yare, and Suffolk Stour (Table 1; Wheeler and Jordan 1990). The redistribution of *B. barbus* in England commenced in the 1890s, when the Hampshire Avon in Southern England had fish introduced from the Thames catchment, with subsequent releases into this river using fish from the Rivers Kennet and Lea. The River Severn had *B. barbus* introduced in 1956 with a release of 509 adult fish from the River Kennet and remains the only known release of fish in either the Severn or its tributary, the River Teme (Table 1; Wheeler and Jordan 1990). This introduction was very successful, and the Severn and Teme have been important recreational fisheries for *B. barbus* since the 1970s. The Warwickshire Avon, also in the Severn catchment, received a stocking of fish in the 1960s from the River Swale (Table 1). This river is highly regulated and it appears unlikely that the river could have been colonized from the Severn due to impassable blockages. Although the focus here was on *B. barbus* in England, the species is also present in Scotland (River Clyde; W. Yeomans pers. comm.) and Wales (River Taff), but samples were not available to the study. The species remains absent from Ireland (Wheeler and Jordan 1990).

**Table 1:** Rivers used in the population genetic study of *Barbus barbus* and details of their catchment, indigenous (I), or nonindigenous (N) range, whether there have been regulated stocking and if so, the dates and source of fish, and the sample size used here. Not included in the table are details on samples analyzed from the River Trent hatchery (*cf*. Materials and Methods)

Pop code	River	Catchment	Range	Stocked	Stocking dates	Source	Sample size
1	Kennet	Thames	I	No			22
2	Thames	Thames	I	Yes	2000s: unknown number of hatchery fish	Trent	20
3	Lea	Thames	I	Yes	2000s: unknown number of hatchery fish	Trent	20
4	Nidd	Yorkshire Ouse	I	Yes	2000s: unknown number of hatchery fish	Trent	7
5	Ure	Yorkshire Ouse	I	No		Trent	9
6	Yorkshire Ouse	Yorkshire Ouse	I	Yes	2000s: unknown number of hatchery fish	Trent	10
7	Wharfe	Yorkshire Ouse	I	Yes	2000s: unknown number of hatchery fish	Trent	14
8	Swale	Yorkshire Ouse	I	Yes	2000s: unknown number of hatchery fish	Trent	3
9	Dove	Trent	I	Yes	2000s: unknown number of hatchery fish	Trent	20
10	Trent	Trent	I	Yes	2000s: unknown number of hatchery fish	Trent	7
11	Great Ouse	Great Ouse	I	Yes	Late 1990s: unknown number 1974: 300 adults 1980s to present: unknown numbers	Kennet Kennet Trent	41
12	Teme	Severn	NI	No			20
13	Severn	Severn	NI	Yes	1956: 509 adults	Kennet	20
14	Warwickshire Avon	Severn	NI		1964: unknown number of adults 2000s: unknown number of hatchery fish	Swale Trent	10
15	Hampshire Avon	Hampshire Avon	NI	Yes	1896: unknown number of adults 1963: 24 adults 1969: 100 adults	Thames Kennet Lea	20
16	Witham	Witham	I^a^	Yes	2000s: unknown number of hatchery fish	Trent	20
17	Wensum	Wensum	I^a^	Yes	2000s: unknown number of hatchery fish	Trent	20
18	Medway	Medway	I^a^	Yes	2000s: unknown number of hatchery fish	Trent	7

a River in the indigenous range but some conjecture over whether *B. barbus* was there naturally (Wheeler and Jordan 1990).

The use of hatchery‐reared *B. barbus* to supplement populations or extend their range in England became more prevalent from the 1980s and continues to present. Fish are usually reared up to the age of 1+ or 2+ years before their release into the wild at lengths of 120–250 mm. Although primarily involving broodstock from the River Trent and completed by Government agencies, some stocking has also been completed using fish cultured in other sites and completed legally by individuals and angling associations, usually using fish of Kennet broodstock (C. Seagrave personal communication). Although these releases were regulated, there is less detail on the numbers of fish released (Table 1). Finally, there is anecdotal evidence suggesting some unregulated movements of *B. barbus* might have occurred between river basins via anglers. There is, however, no documented evidence of this.

### Sampling

In this study, scale samples were available for genetic analyses from 18 rivers in nine river catchments (basins), of which seven catchments were in the indigenous range (but included rivers where it was uncertain if *B. barbus* were found naturally) and two were in the nonindigenous range (Table 1). The scales had been collected from fish sampled either during fish population surveys completed by the Environment Agency (a Government agency in England) between 2001 and 2014, or from fish captured by anglers, with scales removed by a competent person. An exception was scales for the River Great Ouse, where scales were also available from 1994. In all cases, the scales were removed for the purposes of age and growth analysis to support fishery management programmes, rather than specifically for this study. In addition, samples were also available from the River Trent hatchery (10 fish per year from 1997, 2002, 2003 & 2004 and 20 fish from 2000).

### Molecular data

Total genomic DNA was extracted from scales using DNA extraction kit (DNeasy Blood & Tissue Kit and QIAamp DNA Mini Kit, Qiagen), under manufacturer instructions. Phylogenetic and population genetic analyses were performed on (1) mitochondrial DNA Control Region (CR) gene and (2) two nuclear genes (S7 ribosomal protein [S7] and growth hormone [Gh]) (Table 2). These nuclear were selected as suitable markers as they have been extensively used in population studies of barbel species (e.g., Gante et al. 2011, 2015; Meraner et al. 2013; Zaccara et al. 2014; Buonerba et al. 2015). The mtDNA CR was amplified in 268 individuals using primer pair dloop‐sxF and dloop‐dxR (Rossi et al. 2013). As *B. barbus* is tetraploid, the S7 ribosomal protein (S7‐1 and S7‐2) and growth hormone (Gh‐2) genes were amplified using paralog‐specific primers (Gante et al. 2011), methods recently applied in their population genetic analysis elsewhere (Zaccara et al. 2014; Buonerba et al. 2015) (Table 2). Nuclear loci S7‐1 and S7‐2 were successfully amplified with 3–8 and 10–13 primer pairs, while Gh‐2 with 24–30 primer pairs (see Gante et al. 2011). Gh‐1 was not used in this analysis, as we were not able to obtain sequences for a high proportion of the samples. Polymerase chain reaction (PCR) amplifications were performed with Multiplex PCR kit (Qiagen) in 10 *μ*l reaction volume containing approximately 10 ng of template DNA and 0.2 *μ*M of each primer pair. Thermal cycling was performed as follows: denaturation of 15 min at 95°C, followed by 30 cycles of 94°C for 30 sec, 90 sec at 56°C of annealing temperature and the extension step at 72°C for 90 sec, the final elongation was at 72°C for 10 min. The appropriate annealing temperatures were as follows: 58°C for S7‐1, 62°C for S7‐2, and 56°C for Gh‐2 and mtDNA CR. PCR products were purified using kit Illustra^™^ Exostar (GE Healthcare) and sequenced in both directions with amplification primers on an ABI 3130xl Genetic Analyzer using Big Dye 3.1 terminator (Applied Biosystem). The nucleotide sequences of nuclear alleles and CR haplotypes were deposited in GenBank database under accession numbers (KT766197‐KT766290; KT766373‐KT766378) (Table S1).

**Table 2:** Description of samples, including population code (Pop Code), river, and the river catchment (basin). Sample size for molecular analyses, mtDNA CR, growth hormone gene 2 and ribosomal protein (S7), paralog 1 and 2, are also provided

River	Catchment	Pop Code	mtDNA	nDNA		
			CR	Gh‐2	S7‐1	S7‐2
Kennet	Thames	1	17	22	22	22
Thames	Thames	2	19	20	20	20
Lee	Thames	3	20	20	20	20
Nidd	Yorkshire Ouse	4	7	7	7	7
Ure	Yorkshire Ouse	5	7	9	9	9
Yorkshire Ouse	Yorkshire Ouse	6	10	10	10	10
Wharfe	Yorkshire Ouse	7	14	12	14	14
Swale	Yorkshire Ouse	8	3	3	3	3
Dove	Trent	9	20	20	20	20
Trent	Trent	10	7	7	7	7
Great Ouse	Great Ouse	11	15	29	41	41
Teme	Severn	12	18	20	20	20
Severn	Severn	13	20	19	20	20
Warwickshire Avon	Severn	14	10	10	10	10
Hampshire Avon	Hampshire Avon	15	14	17	20	20
Witham	Witham	16	11	13	20	20
Wensum	Wensum	17	20	18	20	20
Medway	Medway	18	7	7	7	7
Hatchery			29	36	60	60
Total			268	299	350	350

### DNA polymorphism

Mitochondrial and nuclear sequences were both manually aligned using BioEdit ver. 5.0.9 (Hall 1999) to eliminate ambiguities and to check polymorphic sites. For the nuclear S7 paralog 2, specimens heterozygous for insertions or deletions (indels) were manually phased using the complementary information carried by the forward and the reverse sequences (Flot et al. 2006). Then, nuclear heterozygous alleles for single nucleotide polymorphism (SNPs) were phased using DnaSP v. 5 (Librado and Rozas 2009); following, the number of haplotypes was calculated using Non Redundant Data Base (Gish 2004). DNA polymorphism indices for each locus, like the number of polymorphic sites (S), the haplotype diversity (Hd), and the percentage nucleotide diversity (*π* %), were calculated using DnaSP v. 5 (Librado and Rozas 2009).

### Phylogenetic analyses

In phylogenetic analyses of mtDNA CR, four sequences from previously sampled individuals of *B. barbus* and *B. plebejus* from Northern Italy (Zaccara et al. 2014) and one available *B. barbus* sequence (GenBank acc. No. AB238965) were added to the dataset, that was rooted with *Barbus meridionalis* (GenBank acc. No. AJ388417).

Phylogenetic analyses were performed on mtDNA CR using three different optimality criteria: maximum likelihood (ML), neighboring‐joining (NJ) and Bayesian analysis. The ML analysis was performed through GARLI v1.0 software (Zwickl 2006) using Trn+I model of sequence evolution (Tamura and Nei 1993), as estimated with ModelTest 3.7 software (Posada and Crandall 1998). Neighboring‐joining was performed trough PAUP 4.0b10 program (Swofford 2002). Bayesian analysis was performed using MrBayes v.3.1.2 software (Ronquist and Huelsenbeck 2003), with a Markov chain Monte Carlo algorithm (MCMC): four simultaneous and independent Markov chains from random trees were started and run for 1,000,000 generations, with the first 25,000 generations (2,500 trees) discarded as the burn‐in (*P* < 0.01).

### Population genetic analyses

A preliminary description of genetic variability at the catchment scale was provided by the mtDNA CR haplotype distribution. Following, each unique nuclear allele was numerically coded and used to genotype each specimen. The genetic variability within populations was quantified (i.e., expected (*H*_E_), observed (*H*_O_) heterozygosity, and all loci were tested for deviation from Hardy–Weinberg equilibrium) using GenePop (Raymond and Rousset 1995; Rousset 2008). In addition, populations were tested for inbreeding by calculating *F*_IS_ using GenePop. Computation of pairwise multilocus *F*_ST_ values (Weir and Cockerham 1984) among populations was performed using Arlequin ver. 3.11 (Excoffier et al. 2005) with 1000 permutation procedure.

Covariation among nuclear loci was assessed using the Bayesian clustering method implemented in the software STRUCTURE v.2.3.4 (Pritchard and Wen 2002) in order to detect the presence of distinct genetic clusters, assign individuals to populations, and to identify migrants and admixed individuals. Each STRUCTURE run consisted of 100,000 MCMC generations as burn‐in, followed by 500,000 MCMC replicates to estimate the posterior sample distribution, using the admixture, correlated allele frequency models. To assess reliability, 20 iterations were run for each K cluster. The number of groups (K) identified by STRUCTURE was estimated by a combination of changes in log‐likelihood of consecutive K‐values and ΔK (Evanno et al. 2005) using the program STRUCTURE HARVESTER (Earl and von Holdt 2012). Results were summarized using CLUMPP (Jakobsson and Rosenberg 2007) and displayed using Distruct (Rosenberg 2004). Finally, in order to better highlight clusters between the populations, a phenetic tree, based on the *F*_ST_ matrix, was built using PAUP 4.0b10 program (Swofford 2002).

## Results

### mtDNA sequence variation at basin scale

**Figure 1:**
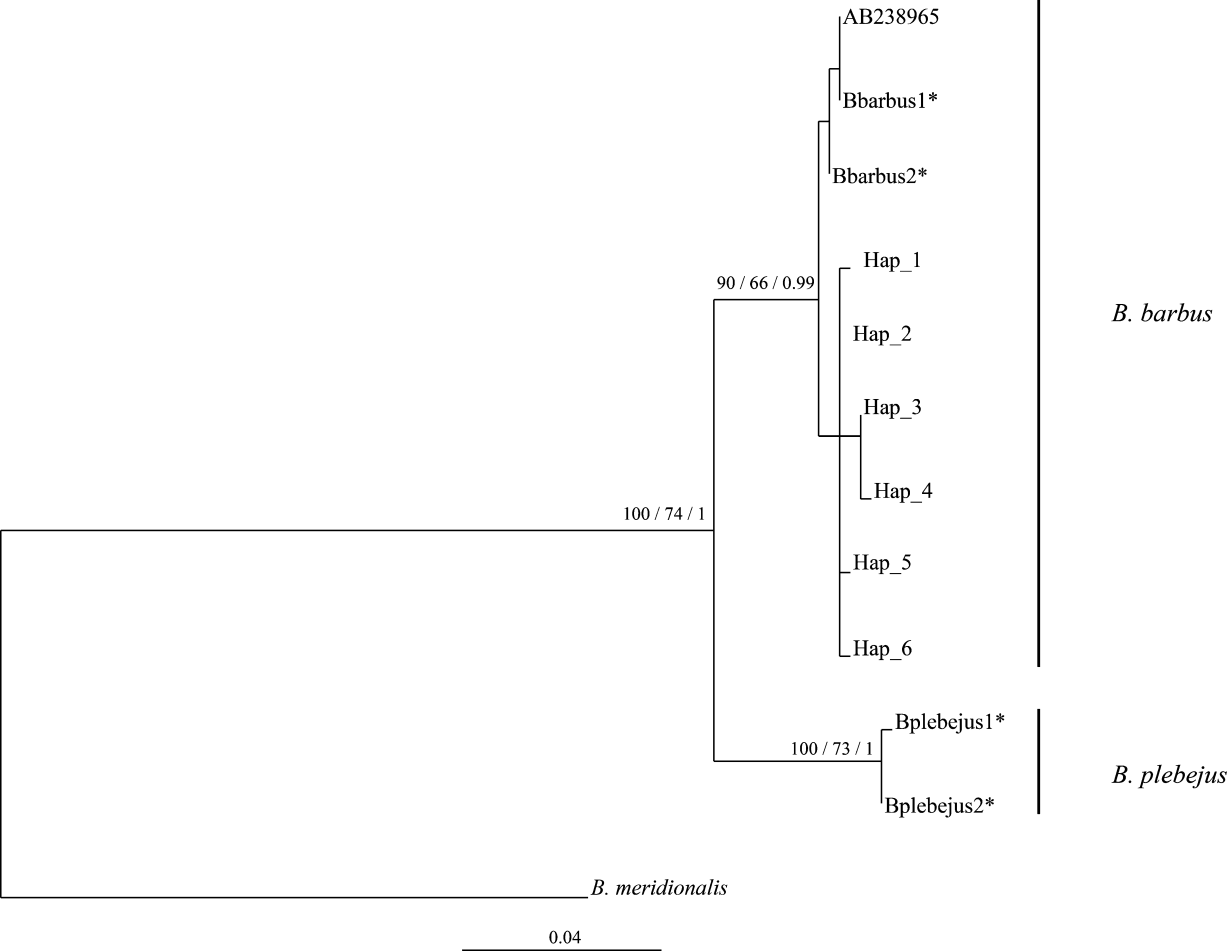
Maximum likelihood (ML) tree based on mtDNA control region (766‐bp length). Node supports are bootstrap values for neighbor‐joining (NJ) and maximum likelihood (ML), and posterior probability for Bayesian inference (BI). Trees were rooted using *B. meridionalis* (AJ388417). *GenBank Accession Number: KT766379 – KT766382.

**Figure 2:**
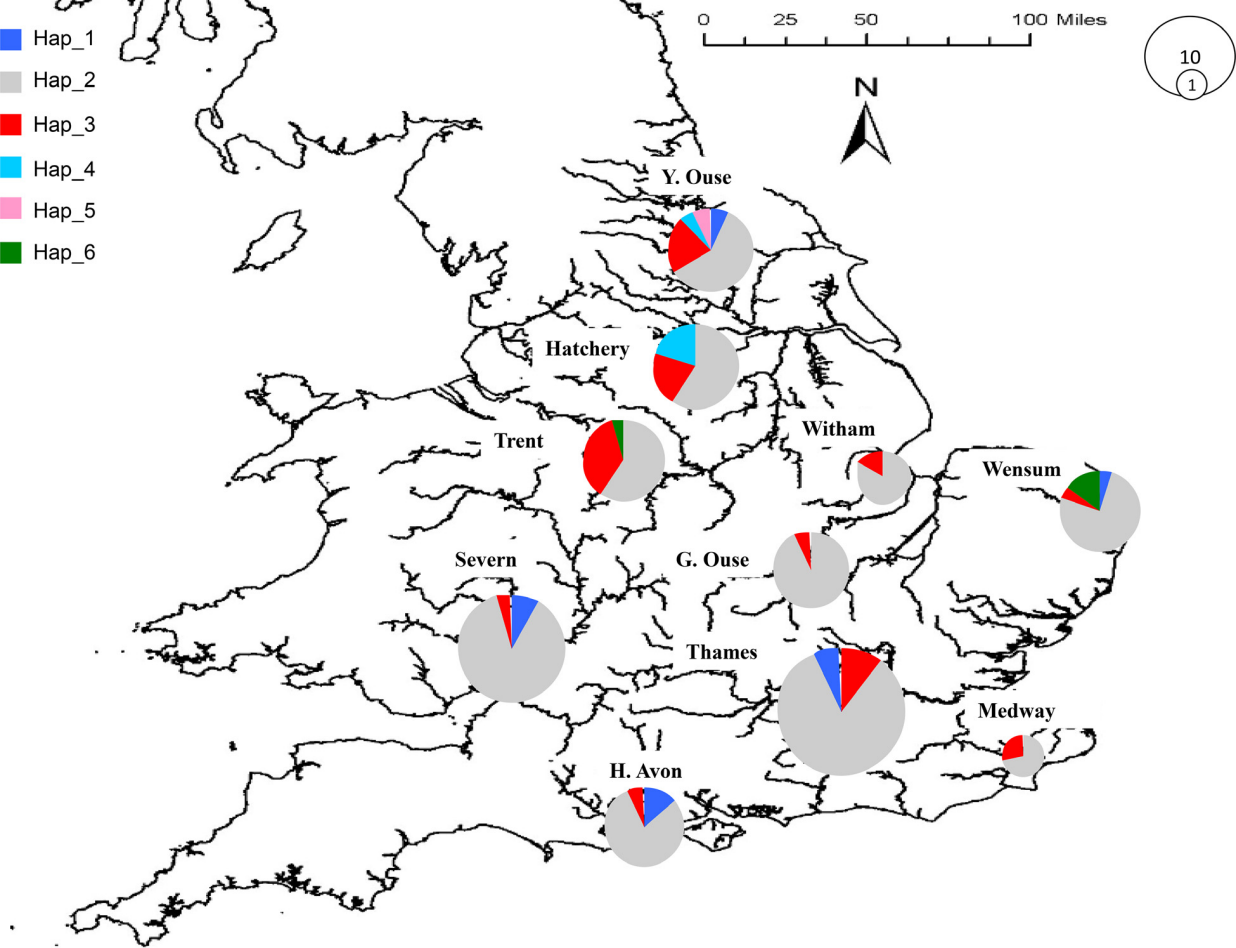
Haplotype distribution between the nine river catchments and the River Trent hatchery, based on mtDNA Control Region (841 bp). The size of circles is proportional to the number of individual fish (see scale).

A total of six different haplotypes were identified in the 941 bp of CR region in the 268 individuals analyzed (Table 2). In the sequence alignment, 6 variable sites were recorded that were all parsimony informative. ML, NJ, and BI phylogenetic analysis of the mitochondrial sequences confirmed that all six UK haplotypes clustered with *B. barbus* (Fig. 1), characterized by weak genetic distance (*P*‐distance = 0.32%), while the interspecies, *B. plebejus,* distance was over 4%. The geographic distribution of the haplotypes revealed a homogenous pattern (Fig. 2). Haplotype Hap_2 was the most abundant, found in 199 individuals and being widespread in all sampled basins and the hatchery. A similar pattern was recorded in haplotype Hap_3 that differed from Hap_2 at 2 nucleotides and was found in all basins (Fig. 2). Haplotype Hap_4 was present only in the Yorkshire Ouse catchment and the hatchery, while Hap_5 was specific to the Yorkshire Ouse catchment only (Fig. 2).

### Nuclear DNA genetic variability

Sequence analysis of three nuclear loci yielded 1998 sequences, with a whole 2058‐bp‐long alignment (S7‐1: 467 bp; S7‐2: 562 bp and Gh‐2: 1029 bp). A total of 1813 SNPs were found, mainly concentrated on the Gh‐2 locus, while only one indel was assumed in the S7‐2 locus. Levels of sequence polymorphism for each marker are summarized in Table S1. Among the 1998 nuclear sequences, 94 haplotypes were scored prevalently expressed by Gh‐2 locus, characterized both by the higher haplotype (H) and by nucleotide (*π* %) diversity (see Table S1).

### Genotyping and population genetic structure analyses

Following genotyping, populations were characterized by *H*_E_ and *H*_O_ values averaged over three loci, ranging from 0.64 to 1.0, respectively; *F*_IS_ values ranged from −0.56 (River Kennet) to −0.14 (River Medway) and were all significant except for two populations (Table 3). These negative values represent an excess of heterozygotes that could be interpreted as lack of inbreeding or genetic drift. Only four populations (Rivers Nidd, Trent, Severn, and Hampshire Avon) had loci that were at Hardy–Weinberg equilibrium.

**Table 3:** Sample size (*N*) and nuclear sequences details for each nDNA marker for genotyping are reported. Tabulated are expected (*H*_E_) and observed (*H*_O_) heterozygosity, results of test for deviation from Hardy–Weinberg (H‐W) equilibrium, average number of alleles (*N*_A_) and estimated fixation indices based on an infinite allele model (*F*_IS_)

Population	Genetic diversity			*F*_IS_	
	*H*_E_	*H*_O_	*N*_A_	Value	Significance
Kennet	0.64	1.00	5.33	−0.56	***
Thames	0.75	1.00	10.67	−0.30	***
Lee	0.65	1.00	5.00	−0.51	***
Nidd	0.74	1.00	6.33	−0.27	*
Ure	0.69	1.00	6.00	−0.39	***
Yorkshire Ouse	0.77	0.97	7.00	−0.21	**
Wharfe	0.74	0.92	7.33	−0.20	**
Swale	0.61	1.00	3.00	−0.50	ns
Dove	0.72	1.00	7.33	−0.36	***
Trent	0.67	1.00	4.33	−0.43	***
Great Ouse	0.69	0.97	9.33	−0.39	***
Teme	0.66	0.98	6.33	−0.46	***
Severn	0.67	0.98	6.33	−0.44	***
Warwickshire Avon	0.67	0.87	5.33	−0.25	**
Hampshire Avon	0.67	1.00	6.00	−0.47	***
Witham	0.64	0.92	6.00	−0.41	***
Wensum	0.70	0.89	8.67	−0.24	**
Medway	0.75	0.90	5.67	−0.14	ns
Hatchery	0.72	0.97	12.33	−0.34	***

Results of permutation testing of significant departure from zero are also given (ns, not significant; *P < 0.05; **P < 0.01; ***P < 0.001).

Among the 171 *F*_ST_ values of pairwise population comparisons, 70 values were significant (*P *<* *0.05). Pairwise *F*_ST_ values that were not significantly different (*P *>* *0.05) were among nonindigenous (NI) populations (Rivers Teme, Severn, Warwickshire Avon and Hampshire Avon), while all values were significant among populations in the indigenous range but where there is conjecture over whether their rivers have natural populations (i.e., Rivers Witham, Wensum and Medway (I*); Table 1). Between the 55 comparisons in the indigenous (I) populations, 21 were significant, mainly in the Yorkshire Ouse catchment (Table 1). Among the 18 comparisons between populations and the hatchery data, 9 were significant, shared out equally among the three population groupings (Table 4).

**Table 4:** Pairwise *F*_ST_ values between populations and their significance (*P *<* *0.05) based on genotyping nDNA markers. The numbers refers to population code. (Populations from 1 to 11 are I, populations from 12 to 15 are NI and populations from 16 to 18 are I*, as defined in Table 1)

Pop code	1	2	3	4	5	6	7	8	9	10	11	12	13	14	15	16	17	18	Hatchery
1	–																		
2	0.15*	–																	
3	−*0.01*	0.10*	–																
4	0.26*	−*0.03*	0.18*	–															
5	*0.02*	*0.05*	−*0.03*	*0.09*	–														
6	0.29*	*0.03*	0.20*	*0.02*	0.09	–													
7	0.31*	*0.01*	0.25*	−*0.02*	0.16*	*0.02*	–												
8	0.49*	−*0.01*	0.37*	−*0.05*	*0.25*	*0.05*	−*0.05*	–											
9	0.13*	−*0.02*	0.09*	−*0.03*	*0.04*	*0.04*	*0.02*	*0.01*	–										
10	0.17*	−*0.04*	*0.11*	−*0.05*	*0.05*	*0.04*	*0.00*	−*0.02*	−*0.04*	–									
11	−*0.01*	0.16*	−*0.01*	0.27*	*0.01*	0.30*	0.33*	0.45*	0.15*	0.19*	–								
12	−*0.01*	0.10*	−*0.01*	0.17*	*0.00*	0.23*	0.24*	0.36*	0.08*	*0.09*	*0.00*	–							
13	*0.01*	*0.04*	−*0.01*	*0.09*	−*0.02*	0.14*	0.16*	*0.21*	*0.03*	*0.03*	*0.01*	−*0.01*	–						
14	*0.02*	*0.03*	−*0.01*	*0.08*	−*0.01*	0.14*	0.15*	*0.22*	*0.03*	*0.03*	*0.01*	−*0.02*	−*0.03*	–					
15	0.02	0.06*	0.00	0.09*	−0.02	0.14*	0.17*	0.24*	*0.04*	*0.04*	*0.03*	*0.01*	*0.00*	*0.01*	*–*				
16	*0.03*	0.12*	*0.01*	0.21*	*0.00*	0.21*	0.27*	0.39*	0.12*	0.16*	*0.00*	*0.03*	*0.02*	*0.01*	*0.04*	–			
17	0.15*	*0.00*	0.11*	*0.02*	*0.08*	0.13*	0.08*	*0.03*	−*0.02*	−0.02	0.16*	0.08*	*0.04*	*0.02*	0.08*	0.14*	–		
18	0.36*	*0.03*	0.28*	*0.00*	0.16*	−*0.01*	*0.00*	*0.01*	*0.04*	0.04	0.37*	0.29*	0.18*	0.16*	0.19*	0.26*	0.13*	–	
Hatchery	0.11*	*0.03*	0.09*	*0.06*	*0.08*	0.20*	0.14*	*0.09*	*0.00*	0.00	0.12*	0.06*	*0.03*	*0.01*	0.08*	0.13*	−*0.01*	0.21*	–

*P *<* *0.05; nonsignificant comparisons in italics.

**Figure 3:**
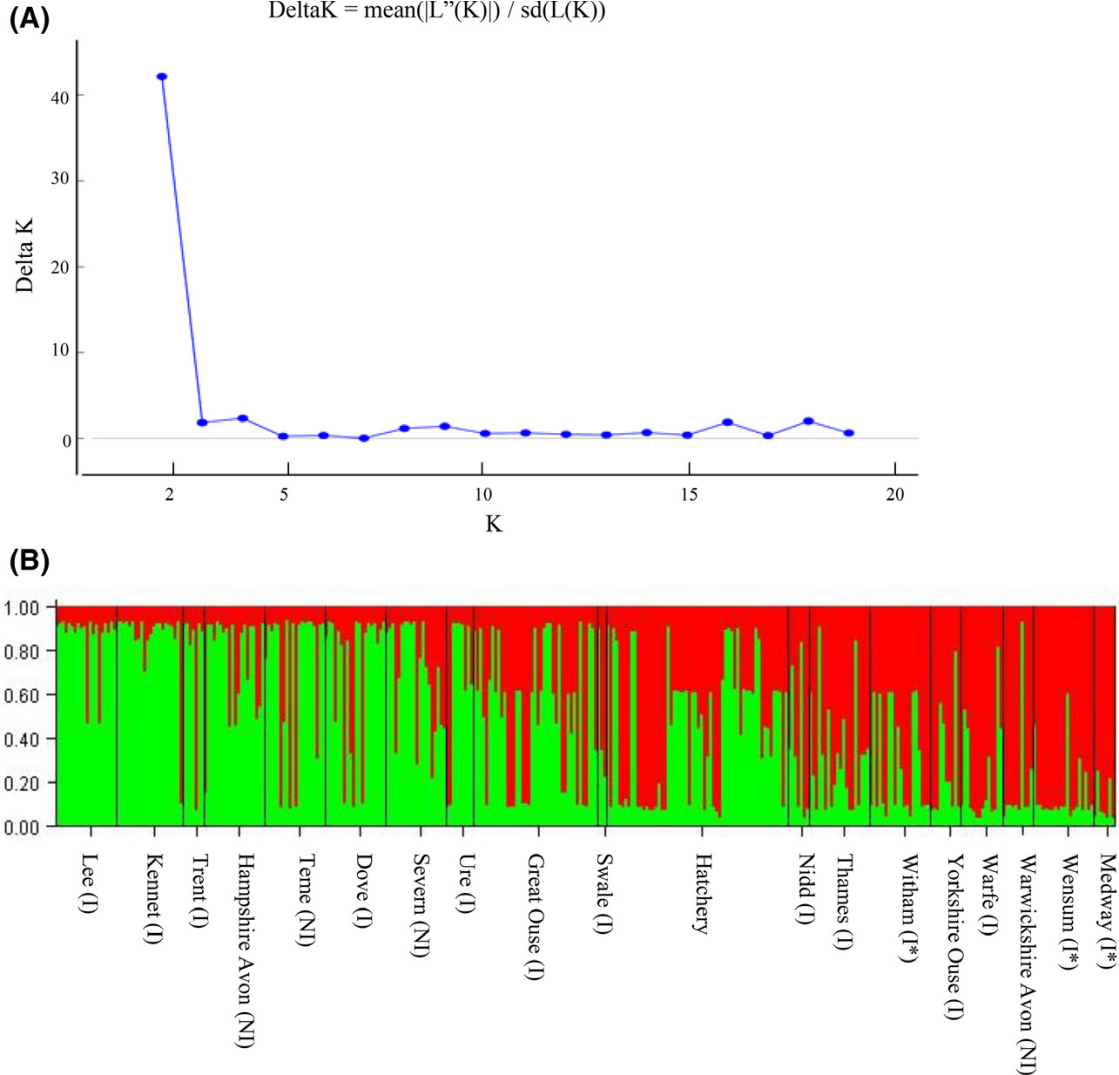
STRUCTURE results: (A) estimate of Δ*K* for each possible values of K using data from STRUCTURE; (B) the STRUCTURE barplot (*K* = 2, highest likelihood run out of 20 repetitions; where red and green denote the two *K* groupings). As defined in Table 1, the distribution range of the population is indicated.

**Figure 4:**
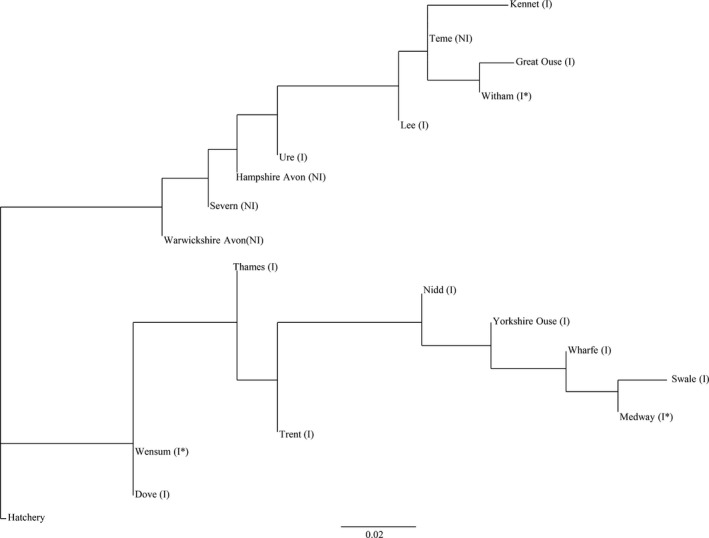
Neighbor‐joining phenetic tree built on *F*_ST_ statistic matrix between populations (see Table 4). Hatchery population was used as an outgroup.

To infer population structure on a finer scale, a STRUCTURE analysis, completed on the entire nDNA dataset, was performed from *K* = 1 to *K* = 20. Using the Evanno et al. (2005) method, two clusters (*K* = 2) were identified and the two statistics used to infer the number of clusters, LnP(D) (−2492.79) and ΔK, were consistent for *K* = 2. The populations were then grouped using their *K* assignment, with a threshold of *K* > 0.7 (Fig. 3). Two river populations (Great Ouse and Swale) and the hatchery population did not reach the *K* > 0.7 threshold. A concordant output was found in the neighbor‐joining phenetic tree, where populations with the same K assignment resulted in the same cluster, with the only exception of four populations (River Witham, Warwickshire Avon, Dove, and Trent) (Fig. 4).

## Discussion

The stocking of *B. barbus* in their indigenous range, and introductions into their nonindigenous range, has been occurring in the UK for over 100 years and these activities continue today even though there is no knowledge of the genetic relationships between the source and recipient populations. This is despite it being well established that understanding genetic differentiation among and genetic variation within populations is important for ensuring measures are implemented to safeguard genetic variability across populations (Dawnay et al. 2008, 2011). For *B. barbus* in England, this has to consider two distinct aspects, genetic impacts in the indigenous range and the influence of invasive populations in the nonindigenous range.

### Indigenous range

The indigenous range of *B. barbus* in the UK only covers some eastern‐flowing rivers in England, a relic of the last glacial period when this landmass was still joined to the European mainland, enabling their colonization by a number of fish species in the late‐glacial and postglacial period from western‐flowing rivers such as the Rhine, Meuse, and Elbe (Wheeler and Jordan 1990). In the *B. barbus* indigenous range in England, two principal groups were identified indicating that the primary separation between these were at river catchment levels: Yorkshire Ouse and Thames, thus representing a north: south pattern of grouping across this range. Indeed, populations in the rivers of the Yorkshire Ouse catchment generally showed relatively high levels of genetic differentiation with populations elsewhere and had at least one specific (unique) haplotype.

The analyses of genetic distance between the populations suggested that policies to enhance populations that involved releasing hatchery‐reared fish have resulted in some genetic‐level homogenization of *B. barbus* populations from different river catchments. For example, across the River Thames catchment, there is significant genetic structuring whereby the Kennet and Lea rivers (both tributary rivers of the River Thames) cluster together but are significantly different from the main River Thames population. Unexpectedly, the main River Thames population is genetically similar to the Yorkshire Ouse and River Trent, possibly due to the introduction of hatchery‐reared fish, with stocking records for the river showing releases in recent years of *B. barbus* from a hatchery that uses River Trent broodstock (Environment Agency, unpublished data). Similarly, the fish from the River Ure (in the Yorkshire Ouse catchment) grouped with the River Kennet and Lee, suggesting that fish were introduced there that originated from these rivers. Losses of population genetic integrity following stocking events have also been detected in populations of salmonid fishes in England when hatchery‐reared fish from other catchments have been released (e.g., Sušnik et al. 2004; Eldridge and Naish 2007). For example, in a study involving 27 UK populations (including England) of grayling *Thymallus thymallus*, while there was considerable population specific genetic diversity evident, it was also revealed that this had been eroded by long‐term stocking of hatchery‐reared fish using broodstock from other catchments (Dawnay et al. 2011).

There are a number of smaller river catchments in this indigenous range in which there is some uncertainty as to whether *B. barbus* populations would have been able to survive there naturally due to, for example, catchment sizes being small and rivers of slow flow that would limit habitat availability and result in small populations vulnerable to environmental changes (Wheeler and Jordan 1990). Such rivers include the Wensum and Medway. In the last 25 years, these small rivers have received stockings of considerable numbers of hatchery‐reared *B. barbus*, particularly using River Trent broodstock, in order to enhance their populations (Environment Agency, unpublished data). The outputs here suggested their populations comprised fish of similar genetic origin. While this could be through the rivers having ancestral populations of similar genetic composition, Wheeler and Jordan (1990) suggested that it was doubtful that these rivers could have historically supported sustainable natural *B. barbus* populations due to their small sizes and variable flow regimes. These rivers have also been subjected to considerable habitat disruptions in the last 50 years through, for example, flood defense works that can substantially affect habitat connectivity and recruitment patterns of cyprinid fishes (Peirson et al. 2008). Consequently, it was considered probable that the similar genetic patterns detected here across these rivers were due more to the hatchery‐rearing stocking activities of recent years rather than their original genetic origins. This then suggests that these stocking activities have been relatively successful with, as a minimum, these resulting in the persistent presence of stocked *B. barbus* in these rivers that enabled their capture and analysis here. This is contrary to many studies on stocking hatchery‐reared fishes of the Cyprinidae family that tend to suggest either their poor survival (e.g., Aprahamian et al. 2004) or relatively low proportions in subsequent samples (Britton 2010). Nevertheless, it does suggest that if there were original, genetically differentiated *B. barbus* populations in these catchments, these have now been lost due to the introgression of the hatchery‐reared fish.

### Nonindigenous range

The translocation of *B*. *barbus* from their indigenous range to their nonindigenous range has been successful, with invasive populations evident in a number of western‐flowing rivers (Wheeler and Jordan 1990; Britton and Gozlan 2013). Commencing over 100 years ago, it originally involved the direct movement of adult fish between catchments, whereas today it is reliant on releasing hatchery‐reared individuals. The genetic data presented here corroborated historical stocking records (Wheeler and Jordan 1990). They revealed high genetic similarity between the indigenous fish of the River Kennet and the nonindigenous fish of the River Severn and Hampshire Avon, where written records suggest the Kennet was the original source of the introduced *B. barbus* (Wheeler and Jordan 1990). For example, the only recorded *B. barbus* translocation into the River Severn was from the Kennet when 509 adult fish were released in 1956. Moreover, these fish have since proved highly invasive by colonizing much of the river and its major tributary, the River Teme, as well as providing a source of fish for translocations into other nonindigenous catchments (Wheeler and Jordan 1990; Britton and Gozlan 2013).

This successful invasion of the River Severn is similar to the invasion success of *B. barbus* observed in other European rivers following introductions. An example is in the River Po basin, Northern Italy, where apparent barriers to migration were unable to prevent the dispersal of invasive *B. barbus* throughout the basin. This was related to intentional releases of fish for angling purposes, resulting in relatively high propagule pressure (Meraner et al. 2013; Zaccara et al. 2014). By contrast, the invasion of the River Severn basin arose from a single founding event, and the invasion of the Hampshire Avon was initiated by an unknown number of fish released in 1896 from the Thames catchment and then 124 fish in the 1960s from the same catchment (Wheeler and Jordan 1990). Thus, these data suggest that in the nonindigenous range in the UK, the release of relatively small numbers of adult fish from either an individual river or a single catchment was sufficient to initiate very successful invasions, with colonization at the catchment level then achieved through natural dispersal and recruitment (Wheeler and Jordan 1990; Britton and Pegg 2011).

In the River Po basin, the *B. barbus* invasion has resulted in population declines of the endemic *Barbus plebejus*, particularly via introgressive hybridization (Meraner et al. 2013; Zaccara et al. 2014). In the invasive UK range of *B. barbus*, there are no other species of the *Barbus* genus present and so their genetic impacts have been negligible. Potential impacts thus relate only to ecological concerns, although these have received little attention to date (Britton and Pegg 2011).

### Evolutionary applications of *B. barbus* genetic data to their population management

Phylogenetic clarification of species assemblages has important implications for conserving and managing populations (Moritz 1994), as unless there is knowledge on what constitutes a species, population or subpopulation, management resources are difficult to assign and strategies difficult to design and implement (Dawnay et al. 2011). Thus, the genetic outputs for *B. barbus* in the UK can be applied to the management of their populations across both their indigenous and their nonindigenous ranges by ensuring management practices that involve fish stocking events always consider their potential for causing detrimental evolutionary consequences in receiving populations.

The mtDNA data revealed *B. barbus* in the UK were within the same clade as populations in mainland Europe that originate in the River Danube catchment, the Western European *B. barbus* glacial refuge (Kotlik and Berrebi 2001). Consequently, they do not constitute a historically isolated unit that could be considered as a single evolutionary significant unit (ESU). However, as inland fish populations tend to be managed within countries, irrespective of their wider ESU status (Dawnay et al. 2011), their populations can then be managed at national levels, with this management informed by their genetic status. For example, as the *B. barbus* of the Yorkshire Ouse catchment (except the River Ure) had at least one specific haplotype and were significantly genetically differentiated from other catchments, it can be argued that they require protection from this being further disrupted by more stockings that originate from outside the catchment. For all other river catchments now containing *B. barbus* in the UK, the genetic data can be applied to either ensuring populations are managed to facilitate the maintenance of their existing levels of genetic differentiation (e.g., the Rivers Kennet, Lea, Severn and Hampshire Avon) or engage in active management to re‐establish populations to their prestocking genetic variability (e.g., River Thames).

Given the lack of overall conservation concern for *B. barbus* across their range, with their IUCN Red Listing being of least concern (IUCN 2015, www.iucnredlist.org), then it can be argued that it remains appropriate that UK populations are managed primarily for recreational, catch‐and‐release angling, especially given the considerable socioeconomic benefits their fisheries can generate (Britton and Pegg 2011). Within this management, measures are thus recommended to either restore or maintain their original genetic diversity, as this should help ensure populations can maintain their ability to adapt to changing environmental conditions, while emphasizing that management approaches should focus more on habitat improvement, such as through improving the longitudinal connectivity of rivers to enable greater access to spawning grounds (e.g., Lucas and Batley 1996; Lucas and Frear 1997) and improving nursery areas (Gordon and Bennetts 1996). Stocking then becomes a last resort to enhance or maintain a population, and given the population structuring detected here, it should focus on only using fish from that river or catchment, especially in the Yorkshire Ouse catchment. In England, this is entirely consistent with current policy and practice for the salmonid fishes brown trout *Salmo trutta* and *T. thymallus* (Environment Agency 2003) and thus also appears highly appropriate for *B. barbus*.

## Conflict of Interest

None declared.

## Data Archiving

Data for this study are available at: *GenBank*.

## Supporting information


**Table S1.** Summary of polymorphisms for S7 paralogs and Gh_2, indicating the number of indels and the number of single nucleotide polymorphisms (SNPs).
