# Loss of genetic integrity and biological invasions result from stocking and introductions of *Barbus barbus*: insights from rivers in England

**DOI:** 10.1002/ece3.2181

**Published:** 2016-05-17

Antognazza C.M., Andreou D., Zaccara S. & Britton J.R.

*Ecology and Evolution* 2016; 6(5): 1280–1292

doi: 10.1002/ece3.1906

The date of the first fish introduction for River Great Ouse should have been in the 1890s, instead of 1990s. The below table has the correct number. This change has no impact in the overall conclusions of the paper. The authors regret this error.

**Table 1:** Rivers used in the population genetic study of *Barbus barbus* and details of their catchment, indigenous (I) or nonindigenous (N) range, whether there have been regulated stocking and if so, the dates and source of fish, and the sample size used here. Not included in the table are details on samples analyzed from the River Trent hatchery (*cf*. Materials and Methods)

Pop code	River	Catchment	Range	Stocked	Stocking dates	Source	Sample size
1	Kennet	Thames	I	No			22
2	Thames	Thames	I	Yes	2000s: unknown number of hatchery fish	Trent	20
3	Lea	Thames	I	Yes	2000s: unknown number of hatchery fish	Trent	20
4	Nidd	Yorkshire Ouse	I	Yes	2000s: unknown number of hatchery fish	Trent	7
5	Ure	Yorkshire Ouse	I	No		Trent	9
6	Yorkshire Ouse	Yorkshire Ouse	I	Yes	2000s: unknown number of hatchery fish	Trent	10
7	Wharfe	Yorkshire Ouse	I	Yes	2000s: unknown number of hatchery fish	Trent	14
8	Swale	Yorkshire Ouse	I	Yes	2000s: unknown number of hatchery fish	Trent	3
9	Dove	Trent	I	Yes	2000s: unknown number of hatchery fish	Trent	20
10	Trent	Trent	I	Yes	2000s: unknown number of hatchery fish	Trent	7
11	Great Ouse	Great Ouse	I	Yes	Late 1890s: unknown number	Kennet	41
					1974: 300 adults	Kennet	
					1980s to present: unknown numbers	Trent	
12	Teme	Severn	NI	No			20
13	Severn	Severn	NI	Yes	1956: 509 adults	Kennet	20
14	Warwickshire Avon	Severn	NI		1964: unknown number of adults	Swale	10
					2000s: unknown number of hatchery fish	Trent	
15	Hampshire Avon	Hampshire Avon	NI	Yes	1896: unknown number of adults	Thames	20
					1963: 24 adults	Kennet	
					1969: 100 adults	Lea	
16	Witham	Witham	I^a^	Yes	2000s: unknown number of hatchery fish	Trent	20
17	Wensum	Wensum	I^a^	Yes	2000s: unknown number of hatchery fish	Trent	20
18	Medway	Medway	I^a^	Yes	2000s: unknown number of hatchery fish	Trent	7

a River in the indigenous range but some conjecture over whether *B. barbus* was there naturally (Wheeler and Jordan 1990).

