# Supplementary material for: Loss of genetic integrity and biological invasions result from stocking and introductions of Barbus barbus: insights from rivers in England
Source: Ecol Evol. 2016 Jan 28;6(5):1280–92. doi: 10.1002/ece3.1906 (PMC4729780; doi:10.1002/ece3.1906)
Supplement: Supplementary file 1 — Table S1. Summary of polymorphisms for S7 paralogs and Gh_2, indicating the number of indels and the number of single nucleotide polymorphisms (SNPs). [file ECE3-6-1280-s001.doc]

Supplementary material

Table AI: Summary of polymorphisms for S7 paralogs and Gh_2, indicating the number of indels and the number of single nucleotide polymorphisms (SNPs). The number of sequences (N), range of sequence lengths (bp), number of haplotypes (h), haplotype diversity (H), percentage of nucleotide diversity (π%), number of segregating sites (S) and GenBank accession number are indicated.

| Nuclear  loci | N | Length  (bp) | h | S | Number  of indels | Total  SNPs | H± sd | π% ± sd | GenBank  Accession number |
| --- | --- | --- | --- | --- | --- | --- | --- | --- | --- |
| *S7_1* | 700 | 467 | 2 | 2 | - | 150 | 0.255 ± 0.019 | 0.109 ± 0.008 | KT766197 - KT766198 |
| *S7_2* | 700 | 559 - 562 | 10 | 12 | 1 | 183 | 0.250 ± 0.021 | 0.123 ± 0.011 | KT766199 - KT766208 |
| *Gh_2* | 598 | 1029 | 82 | 21 | - | 1480 | 0.898 ± 0.007 | 0.652 ± 0.012 | KT766209 - KT766290 |
| Total | 1998 |  | 94 |  |  | 1813 |  |  |  |
